# Phonon-mediated generation of quantum correlations between quantum dot qubits

**DOI:** 10.1038/srep23753

**Published:** 2016-04-01

**Authors:** Jan Krzywda, Katarzyna Roszak

**Affiliations:** 1 Department of Theoretical Physics, Faculty of Fundamental Problems of Technology, Wrocław University of Technology, 50-370 Wrocław, Poland

## Abstract

We study the generation of quantum correlations between two excitonic quantum dot qubits due to their interaction with the same phonon environment. Such generation results from the fact that during the pure dephasing process at finite temperatures, each exciton becomes entangled with the phonon environment. We find that for a wide range of temperatures quantum correlations are created due to the interaction. The temperature-dependence of the level of correlations created displays a trade-off type behaviour; for small temperatures the phonon-induced distrubance of the qubit states is too small to lead to a distinct change of the two-qubit state, hence, the level of created correlations is small, while for large temperatures the pure dephasing is not accompanied by the creation of entanglement between the qubits and the environment, so the environment cannot mediate qubit-qubit quantum correlations.

Quantum correlations play a crucial role in the understanding and possible implementation of any quantum computation algorithm^1^,^2^,^3^. Unfortunately the influence of the environment is usually hostile to entanglement^4^,^5^,^6^, which is the standard type of quantum correlations used for quantum computation. It has been recently shown that a weaker type of quantum correlations, those which are measured by the quantum discord^7^,^8^,^9^ and which are sometimes present in separable (nonentangled) states, are also useful from the perspective of quantum computation^10^,^11^,^12^,^13^,^14^,^15^,^16^,^17^,^18^,^19^. Typically, an interaction with the environment is also detrimental to the quantum discord, but the discord is expected to be much more robust under the influence of the environment, and under some conditions may even be enhanced by noise^9^.

Quantum dot (QD) excitonic qubits^20^, for which one of the qubit states is an empty QD and the other is a ground state exciton excited in the QD, unavoidably interact with a bath of vibrations of the lattice of the crystal in which the dot is embedded (phonon environment)^21^,^22^,^23^,^24^,^25^. This interaction is diagonal in terms of QD states and hence, can only lead to pure dephasing of excitonic qubits. Contrarily to interactions with environments which are not diagonal in the subspace of qubit eigenstates, some of which have been shown to lead to the generation of inter-qubit entanglement^26^,^27^,^28^, the interaction does not lead to entanglement between the qubits. Yet it has been recently shown that such a process is, at finite temperatures, always accompanied by the creation of entanglement between the qubit and the environment^29^, and hence, we can expect some kind of quantum correlations to be generated between qubits via the interaction with a common phonon environment.

We study a system composed of two QD excitonic qubits separated by a finite distance and, hence, interacting with a common environment of phonons. We find that such an interaction will lead to the creation of finite quantum discord values between the two qubits, if the distance between them is small enough that the environments cannot be treated as separate, and the temperature is modest. Because of the characteristic “partiality” of phonon-induced processes, the generated discord is robust until the influence of other, slower decoherence mechanisms becomes dominant. We identify two most prominent features of the evolution during the generation of the quantum discord and study their origin and parameter dependence (which are both different) with the help of X-states whose quantum correlations are easier to quantify.

## Results

### Generation of quantum correlations

The system under study consists of two QD excitonic qubits, where the |0〉 and |1〉 qubit states denote the empty dot and the single exciton in its ground state, respectively. Such excitonic qubits are known to suffer from a strong interaction with an environment of phonons (quanta of the vibrations of the crystal lattice in which the dots are embedded)^21^,^22^. The interaction leads to pure dephasing of the qubit states which is only partial (the qubit coherences decrease up to some finite value which is strongly dependent on temperature) due to the super-Ohmic nature of the phonon bath. As has been recently shown in ref. 29, the dephasing process is accompanied by the creation of entanglement between the qubit and its phonon environment. Since the two QDs interact with the same phonon environment (unless they are infinitely distant from each other) it is reasonable to expect a generation of quantum correlations between the two qubits which would result from the fact that both of them become entangled with a common bath.We study the evolution of quantum correlations between two qubits in a pure initial state which is not correlated in any way (quantumly or classically). To this end we assume that both qubits are in the same pure initial state given by 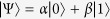, where 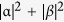 =1. The initial two-qubit state is a product of the two single qubit states and is then given by


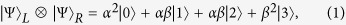


where the indices *L* and *R* differentiate between the two dots on the left side of the equation and a two-qubit basis is used on the right side with 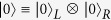, 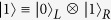, 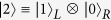, and 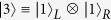. Obviously the state is separable and it also has zero discord.

We also assume that the whole system, consisting of the two qubits and the phonon environment, is initially in a product state, and that the environment is then at thermal equilibrium. The Hamiltonian of this system and the Weyl operator method which allows for the exact diagonalization of such Hamiltonians are presented in the Methods Section. The exact formulas which govern the evolution of the two QDs after the degrees of freedom of the environment are traced out are also explicitly stated there together with a detailed discussion of the behavior of the two qubit density matrix in general and in the long-time, high-temperature, and large-distance between the dots limits.

In the following we do not take into account the interaction between the qubits (the biexcitonic shift which describes the energy shift of the state |11〉 due to the interaction when excitons are present in both dots). This is because the presence of the biexcitonic shift does not change the level of correlations generated between the qubits due to the interaction with the environment, while it leads to oscillations between the initial separable state and the maximally entangled state 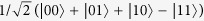. In the presence of phonons these oscillations are damped while finite periods of time appear when entanglement is equal to zero^6^ (sudden death type behaviour followed by the rebirth of entanglement). In the case of the quantum discord, the same oscillations are seen, but phonon dephasing leads to characteristic indifferentiable behavior near the state with highest entanglement, while discord curves are smooth around the zero-discord states^30^.

It turns out that the described process can only lead to emergence of inter-qubit entanglement in extreme situations, for which the number of phonon modes taken into account is severely restricted and the exciton-phonon coupling is extremely high. In all realistic situations, to which we limit ourselves here, the qubit-qubit interaction mediated by qubit-phonon entanglement can only lead to the appearance of weaker quantum correlations described by the quantum discord (while the two-qubit state remains separable). Nevertheless, quantum correlations between qubits do appear due to the process of phonon-mediated decoherence.

The measure of quantum correlations used in the following is the rescaled discord^31^. This is a geometric measure which is related to the geometric discord. The geometric discord is defined as the smallest Hilbert-Schmidt distance between the studied state and the set of zero-discord states^32^ and is one of the few discord measures which can be evaluated from the two qubit density matrix for any two qubit state. In fact, explicit formulas are known for the upper^33^ and lower^32^ bound on the geometric discord. The transition to the rescaled discord (which can be easily found for a pair of qubits, if the geometric discord is known) is made to ensure that the measure does not depend on the purity of the state (as the Hilbert-Schmidt distance does). The exact details needed for the calculation of the bounds on the geometric discord and the rescaled discord are given in the Methods Section.

**Figure 1:**
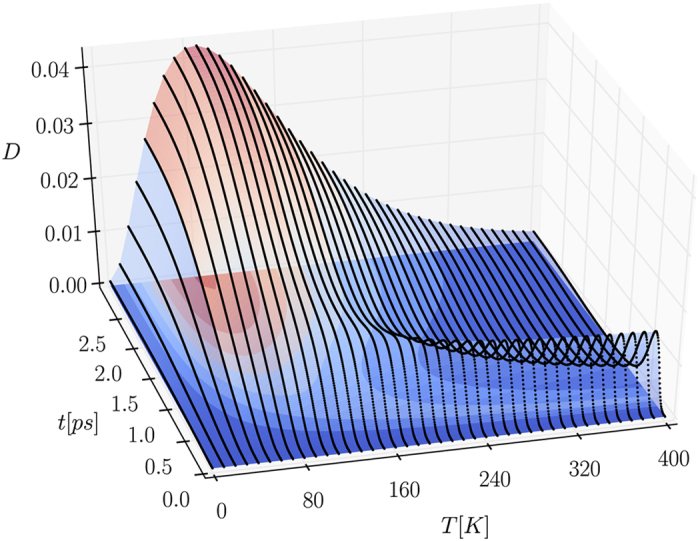
Evolution of the rescaled discord as a function of time and temperature for the initial two-qubit state (1) with 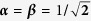 at the distance between dots *d* = 6 nm.

Figure 1 shows the evolution of the rescaled discord (the lower and upper bounds of the discord coincide up to numerical precision for the data shown) of the initial state (1) with 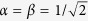 as a function of time and temperature with the distance between the dots is set at *d* = 6 nm (the studied dots lie on the same plane, are assumed identical, and are small and flat, with the height of the dots set as 1 nm and the width around 4 nm; the width of the wavefuncitons describing the electron and hole are slightly different). In fact, the discord upper and lower bounds always coincide when *α* = *β*; this is not the case when *α* ≠ *β*. As can be seen, the interaction with a common phonon environment does lead to the appearance of quantum correlations between the two QDs. This is due to the fact that for any finite temperature the exciton-phonon interaction leads to entanglement between each qubit and the phonon environment as shown in ref. 29 (there is no time delay between the start of the joint exciton-phonon evolution and the appearance of entanglement). Since the dots are coupled to the same environment (for any finite distance between them *d*), this allows for phonon-mediated transfer of quantum correlations.

There are two prominent features of the generation of phonon-induced quantum correlations which are both visible in Fig. 1. The first is the appearance of a maximum at short times (sub-picosecond). The second is the accumulation of quantum correlations at slightly longer times (on the order of picoseconds) which leads to the emergence of a plateau. The effect of temperature on the plateau (in terms of its height and the time when it is reached) is much stronger than the effect of temperature on the maximum and when the maximum is visible it survives for higher temperatures than the plateau. Both features are small at low temperatures, do not occur at infinite temperatures (since no exciton-phonon entanglement is created), and reach their maximal values at some finite temperatures (which are different for the two features). Contrarily, the effect of the distance between the dots is much stronger on the maximum and the maximum is visible only for very closely spaced QDs (the discord values at the maximum and at the plateau both grow with the inverse of the interdot distance).

**Figure 2:**
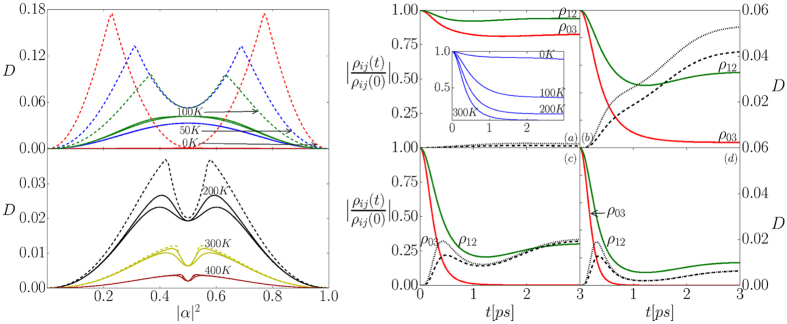
Left: Long-time (steady state) discord dependence on the single qubit occupation |*α*|^2^ at different temperatures. Solid lines correspond to the pure initial state (1) and dashed lines correspond to the X initial state (2). Top panel contains curves for *T* ≤ 100 K and bottom panel contains curves for *T* > 100 K. Right: Evolution of normalized coherences at **(a)** 0 K, **(b)** 100 K, **(c)** 200 K, (**d**) 300 K. Red line - *ρ*_03_, green line - *ρ*_12_. Inset - *ρ*_01_. The evolution of the rescaled discord corresponding to the coherences is given by the dashed black lines for initial pure state (1) with 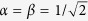 and by the dotted black lines for the respective initial X-state (2) with *a* = *b* = *c* = *x* = *y* = 1/4.

To understand the origin of the two features and the nature of their parameter dependences, it is necessary to understand what (phonon-induced) changes to the DQD density matrix cause them to emerge. To this end, the evolutions of the amplitudes of the normalized off-diagonal elements of the density matrix |*ρ*_*ij*_(*t*)|/|*ρ*_*ij*_(0)| are plotted in the right panel of Fig. 2 for different temperatures. The amplitude values corresponding to the elements *ρ*_03_(*t*) (describing the coherence between |00〉 and |11〉; red solid lines) and *ρ*_12_(*t*) (describing the coherence between |01〉 and |10〉; green solid lines) responsible for inter-qubit coherence are expected to be most important for the generation of quantum correlations. The amplitudes of all other coherences follow the same decay function which is plotted in the inset of the right panel of Fig. 2(a). The decay of the three curves corresponding to different off-diagonal elements differs substantially. The decay of *ρ*_03_(*t*) is faster than the decay of the other curves, since it is the coherence between states which are easiest to distinguish for phonons (the state when both dots are empty and the state when two excitons are excited). The coherences between states that differ by one exciton (*ρ*_01_(*t*), *ρ*_02_(*t*), *ρ*_13_(*t*), and *ρ*_23_(*t*)) evolve more slowly, but display the same type of decay as the *ρ*_03_(*t*) curve. The element *ρ*_12_(*t*) describes the coherence between states which globally have one exciton which is either in the left or in the right dot, so they are the hardest for phonons to distinguish (especially for small distances between the dots). Hence, the decay of this coherence qualitatively differs from all other coherences and it shows a slight revival at finite times which is due to the interference of wave-packets from different QDs.

The dashed black lines in the right panel of Fig. 2 show the evolution of the rescaled discord for initial state (1) with 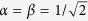 (same as in Fig. 1) corresponding to the decoherence curves shown in the same figure. Careful examination reveals that the initial maximum corresponds to the fast decay of the *ρ*_03_(*t*) curve with respect to the deacay of *ρ*_12_(*t*). This is followed by a rise of the quantum discord until it reaches a plateau which corresponds to the revival of the *ρ*_12_(*t*) curve and it also reaching a plateau.

Further analysis of the processes which lead to the generation of quantum correlations here requires a simplified scenario for which the quantum discord can be calculated analytically. Since the situation under study involves only pure dephasing, an initial X-state will remain an X-state throughout the evolution. For an X-state, which is generally of the form


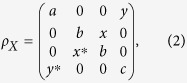


the lower and upper bounds on the geometric discord coincide, and the value of the geometric discord can be easily found using the formulas for the lower bound on the geometric discord given in the Methods Section. For 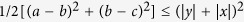 we get


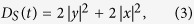


while for 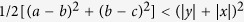, the geometric discord is given by


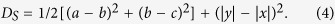


If 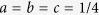 and the initial X-state differs from the previously studied pure initial state only by four coherences which are set to zero, meaning that *a*(0) = |*α*|^4^, 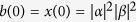, *c*(0) = |*β*|^4^, and *y*(0) = *α*^2^β^*2^, then the geometric discord is in the regime described by Eq. (4) at all times and is in fact equal to *D*_*S*_ = (|*y*| − |*x*|)^2^. Obtaining the rescaled discord now only requires inserting this equation into Eq. (8) as discussed in the Methods Section.

The rescaled discord for the initial state (2) with a = b = c = x = y = 1/4 (which resembles the pure, equal superposition state of Eq. (1) with 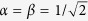 the closest of all X-states) evolving in the same way as the equal superpositon state would is plotted in the right panel of Fig. 2 with black dotted lines. The strong resemblance of the discord evolution of this X-state and the corresponding pure state is striking. Including the presence and the evolution of the four off-diagonal elements missing in the X-state changes only the quantitative features of the discord evolution (the discord generated in the pure state is slightly smaller), but the qualitative features remain the same. This means that the appearance of the maximum and the plateau is of the same origin for the two states and that it is the difference of the amplitudes of the two two-qubit coherences that is crucial for the phonon-induced generation of quantum correlations between qubits. Note, that the appearance of the maximum stems from the difference in the rates of the initial dephasing of the two off-diagonal elements and as such the process could not lead to the appearance or enhancement of entanglement. This process does not in fact require generation of qubit-environment entanglement for the appearance of non-zero inter-qubit quantum discord to be possible^9^, which explains its much weaker temperature dependence than that of the plateau. The plateau, on the other hand, is related to the revival of the *ρ*_12_ coherence, which is a common feature in few-qubit phonon decoherence, and is connected with the interference of phonon wave-packets travelling away from the dot. It can also lead to the revival of entanglement as seen in ref. 6.

Finally, the study of the X-state resolves the problems of the dependence of the generated quantum discord on temperature and distance between the dots. Firstly, for low temperatures, since there are few phonons in the system the resulting pure dephasing is weak, so the difference between the two coherences is small and so is the value of the quantum discord. For large temperatures, both coherences are strongly affected (and the revival effect becomes negligible) which diminishes the difference between them, again leading to small values of the quantum discord plateau (and to zero discord for infinite temperatures). Hence, the discord which is generated at the plateau is relatively high only for intermediate temperatures for which phonon effects are already strong while qubit-environment entanglement is still effectively created. The maximum survives to higher temperatures, since it does not require the build-up of quantum correlations. As for the dependence on the distance between the quantum dots, the difference in evolution between |*ρ*_03_| and |*ρ*_12_| stems from the fact that both QDs interact with the same phonon environment. When the dots are infinitely far apart (separate phonon environments) the relation |*ρ*_03_| = |*ρ*_12_| is fulfilled at all times leading to no quantum correlations ever being generated. Both the difference between the rates of dephasing of *ρ*_03_ and *ρ*_12_ and the magnitude of the revival of *ρ*_12_ decrease with growing distance between the dots (the time of revival grows with this distance) while the environment with which they each interact becomes different. The plateau is much more robust against growing distance between the dots than the maximum, since in the initial stages of decoherence (for short times) the evolution reaches the limit where it behaves similarly to the situation when each dot interacts with a separate environment for much smaller distances than in the later stages (for longer times). As distance grows, the plateau appears later in time (since it takes phonons from the two QDs longer to reach each other), but it does appear for all reasonable distances between the dots.

Lastly, let us study the dependence of the generated quantum correlations on the initial single qubit state. To this end, long time (steady state) rescaled discord of the initial state given by Eq. (1) is plotted as a function of single qubit occupation |*α*|^2^ (for *α* = |*α*| and 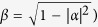 in the plot on the left side of Fig. 2 (solid lines) at different temperatures. The first important feature is that for *α* ≠ *β* the lower and upper bounds on the discord do not necessarily coincide (both bounds are plotted in the figure). Secondly, for higher values of temperature (for which the dephasing is strong) the discord is not a convex function of |*α*|^2^ (lower panel of the figure), which could be expected and is true for lower temperatures (upper panel of the figure).

The dependence of the long-time rescaled discord on |*α*|^2^ for the initial state (2) with *a* = |*α*|^4^, *b* = *x* = *y* = |*α*|^2^(1 − |*α*|^2^) and *c* = (1 − |*α*|^2^)^2^ is also shown in the plot on the left side of Fig. 2 (dashed lines). The comparison of the asymptotic discord of such states to that of the corresponding pure states is more tricky than in the case of 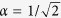, because only for *α* = 0, 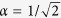, or *α* = 1 the discord of the initial state is equal to zero (so only then there are no quantum correlations present in the initial state). Otherwise the initial discord value is finite and the initial geometric discord is obtained using Eqs (3) and (4), which yield


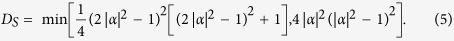


The long-time values of the X-state discord clearly show the transition between different regimes of decay (here as a function of |*α*|^2^) which is characteristic of the discord^30^,^34^,^35^,^36^. The transition occurs when the dependence of the discord as a function of |*α*|^2^ changes between increasing and decreasing (on the left plots in Fig. 2). Note, that it occurs irrelevant of the temperature studied. For low temperatures (the upper panel of the plot on the left side of Fig. 2) there is no resemblance in the |*α*|^2^-dependence between the pure-state (1) and the X-state (2). This is because at low temperatures the four coherences which are initially zero for the X-state are preserved at reasonably large values in the pure state evolution. This is not the case for high temperatures and the level of long-time correlations present in the initially pure two qubit system starts to resemble that of the X-state. Although there is no visible transition between discord decay regimes for the initial pure states, the dependence on the single qubit occupation shows a similar pattern as that of the X-state. This is because for high temperatures the “irrelevant” coherences are quickly and largely reduced by the interaction with phonons and the pure state qualitatively resembles the X-state at long times.

## Discussion

We have studied the generation of quantum correlations between two QD qubits which do not interact directly, but are coupled to a common phonon bath. Such a qubit-environment interaction leads to the generation of entanglement at finite temperatures. As we have shown, this suffices for the generation of quantum correlations described by the quantum discord (but not entanglement itself) between the qubits. Since phonon-induced decoherence is only partial, the long-time correlations generated are robust (until other slower decoherence sources, such as exciton recombination, take over).

The time-evolution of the two-qubit quantum discord of an initial pure product state displays two main features which depend in a non-trivial manner (and differently) on temperature and interdot distance. The features result from different decoherence mechanisms and while the initial feature (maximum) could be caused by noise which does not result from the generation of qubit-environment entanglement the second feature (plateau) stems from the same process which leads to the slight rebirth of two-qubit coherence and can reduce two-qubit entanglement decay. Hence, the plateau mechanism which is the one leading to robust discord generation requires entanglement between the qubits and their common environment.

Finally, we have studied the dependence of the level of quantum correlations generated on the initial single qubit occupations. This dependence becomes counterintuitive beyond some threshold temperature (around 150 K for the studied system) and instead of displaying monotonous behavior when the occupation changes from completely asymmetric (|*α*|^2^ = 0 or |*α*|^2^ = 1) to fully symmetric (|*α*|^2^ = 1/2), we observe a maximum at some value of |*α*|^2^ between zero and one half and a local minimum is reached at |*α*|^2^ = 1/2. The origin of this behavior can only be understood with the help of corresponding X-states (which is reasonable at high temperatures), the study of which reveals that the non-monotonicity is related to transitions between different decay regimes which is characteristic for quantum discord evolutions.

## Methods

### Double quantum dot evolution in the presence of phonons

The system under study consists of two excitonic QD qubits located in one plane and interacting with a common phonon environment^21^,^22^. The basis states of each qubit are |0〉 which corresponds to an empty QD and |1〉 which denotes an exciton in its ground state excited in the QD. The system is described by the Hamiltonian *H* = *H*_*L*_ + *H*_*R*_ + *H*_*ph*_, where 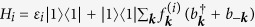, with *i* = *L, R* differentiating the dots, describes the energy of each dot and its interaction with the phonon environment. The third part of the Hamiltonian describes the free phonon energies 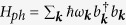. Here, *ε*_*i*_ are the transition energies in the two subsystems, 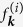 are system-environment coupling constants, 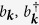 are bosonic operators of the phonon modes, and 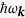 are the corresponding energies. The energy shift due to the interaction between the subsystems (the biexcitonic shift) has been omitted here, since it leads to coherent two-qubit rotations and consequently to oscillations of entanglement that would only distort the phonon-induced generation of quantum correlations that we want to observe.

This type of Hamiltonian can be diagonalized analytically following ref. 6 by the transformation 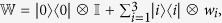 where in the two-qubit basis the states denote 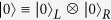, 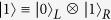, 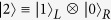, and 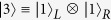. The operators *w*_*i*_ are Weyl shift operators and are given by 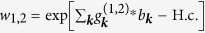 and 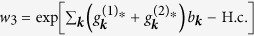, where 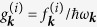. The diagonalized Hamiltonian is given by 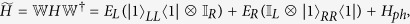 where the left side in the tensor product corresponds to the left dot (*L*) and the right side to the right dot (*R*). The shifted energies here are given by 
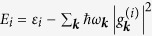
.

The evolution operator of the whole system may now be written in terms of the evolution operator of the diagonalized Hamiltonian and Weyl operators as 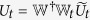, where 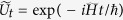 and 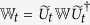. Hence, when the system and environment are initially in a product state, with the environment in a thermal equilibrium state *ρ*_*T*_, the reduced density matrix of the two-qubit system is given by 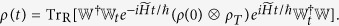 Here 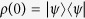 denotes the initial density matrix of the two qubits. The phonon induced evolution results in pure dephasing, meaning that the diagonal elements of the DQD density matrix remain constant. Using the rules for multiplying and averaging Weyl operators^37^ one finds the evolution of the off-diagonal elements of the DQD density matrix,


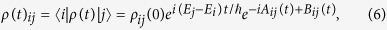


with *E*_0_ = 0, *E*_*1*_ = *E*_*L*_, *E*_*2*_ = *E*_*R*_, and *E*_3_ = *E*_*L*_ + *E*_*R*_. For two QDs on the same plane (not on top of each other) the decoherence is governed by the functions


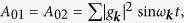



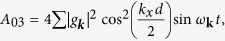



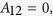



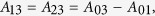



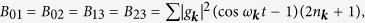



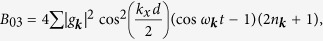



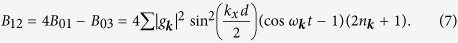


Here 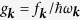 and the individual dot coupling constants were taken to be 
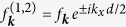
, meaning that the exciton-phonon coupling is the same for each dot and they are displaced by the distance *d* along the *x* axis (in-plane). For long times, the factors cos *ω*_***k***_*t* become quickly oscillating functions of ***k*** and their contribution averages to 0. Consequently, *B*_*ij*_ decrease form their initial value of 0 to a certain asymptotic value depending on the material parameters, system geometry and temperature. For large distances between the dots *d*, on the other hand, the distance-dependent factors cos^2^(*k*_*x*_*d*/2) and sin^2^(*k*_*x*_*d*/2) become quickly oscillating and their contribution averages to 1/2 which leads to *B*_03_ = *B*_12_ = 2*B*_01_ and *A*_03_ = 2*A*_01_. In this case the evolution of each qubit can be described separately and the single qubit coherence decays following Eq. (6) with *A*_01_ and *B*_01_, which concurs with the fact that distant QDs interact with practically separate environments.

The results shown in the previous sections have been obtained using the following parameters. The exciton wave functions have been modeled by anisotropic Gaussians with the extension *l*_e/h_ in the *xy* plane for the electron/hole, and *l*_*z*_ along *z* for both particles. The coupling constants for the deformation potential coupling between confined charges and longitudinal phonon modes then have the form 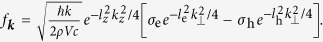 Here *V* is the normalization volume of the bosonic environment, *k*_⊥_, *z* are phonon momentum components in the *xy* plane and along the *z* axis, *σ*_e/h_ are deformation potential constants for electrons and holes respectively, *c* is the speed of longitudinal sound, and *ρ* is the crystal density. In our calculations we put *σ*_e_ = 8 eV, *σ*_h_ = −1 eV, *c* = 5.6 nm/ps, *ρ* = 5600 kg/m^3^ (corresponding to GaAs), and *l*_e_ = 4.4 nm, *l*_h_ = 3.6 nm, *l*_*z*_ = 1 nm. These parameters correspond to small self-assembled QDs^38^,^39^.

### Rescaled Discord

To measure the quantum correlations generated between the two qubits we used a geometric measure of the quantum discord called the rescaled discord^31^. The rescaled discord is related to the geometric discord^32^, which is defined as the Hilbert-Schmidt distance between a given state and the nearest zero-discord state, but it is independent of the purity of the studied state. For two qubits the relation between the discord measures is


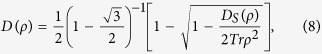


where *D* denotes the rescaled discord, *D*_*S*_ the geometric discord and Tr*ρ*^2^ is the purity of the two qubit density matrix.

For two qubits, it is possible to find the lower and upper bounds of the geometric discord (and consequently the rescaled discord) given the density matrix of the state. The lower bound on the geometric discord is given by^32^ 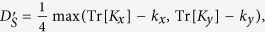 where *k*_*x*_ is the maximum eigenvalue of the matrix 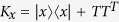 and *k*_*y*_ is the maximum eigenvalue of the matrix 
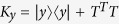
. Here, |*x*〉 and |*y*〉 denote local Bloch vectors with components 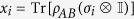 and 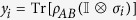, and the elements of the correlation matrix *T* are given by 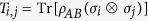 (stemming from the standard Bloch representation of a two-qubit density matrix *ρ*_*AB*_). The upper bound is given by^33^ 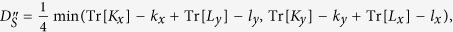 where *l*_*x*_ and *l*_*y*_ are the maximal eigenvalues of the matrices 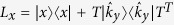 and 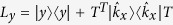, respectively, while 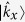 and 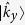 are the normalized eigenvectors corresponding to the eigenvalue *k*_*x*_ of matrix *K*_*x*_ and *k*_*y*_ of matrix *K*_*y*_. The final step in acquiring the upper and lower bounds on the rescaled discord is inserting the geometric discord values into Eq. (8). For two qubits the rescaled discord can vary between zero and one half, where 0 indicates no quantum correlations present between qubits and 1/2 is reserved for maximally entangled states (the biggest possible quantum correlations).

## Additional Information

**How to cite this article**: Krzywda, J. and Roszak, K. Phonon-mediated generation of quantum correlations between quantum dot qubits. *Sci. Rep.* **6**, 23753; doi: 10.1038/srep23753 (2016).
